# Methods of Consolidating Gold Fillings

**Published:** 1871-07

**Authors:** S. G. Perry

**Affiliations:** New York.


### ARTICLE VII.

### *Methods of Consolidatiny Gold Fillings.*

By S. G. Perry, D. D. S., New York.

Read before the New York Odontological Society, April 18, 1871

In considering the consolidation of gold, I shall be obliged to give some attention to the preparation of cavities and to the kind of gold used. Without discussing the principles involved or considering the methods adopted by others, I can only give the conclusions at which I have arrived through my own experience. Perhaps this can be done in fewest words by a simple description of my own method.

The *best* operations I ever performed were made with adhesive gold, packed with small-pointed, finely-serrated, nearly straight instrument, in cavities easy of access, free from under-cuts, and in teeth around which the rubber dam could be used. Here, then, I have a criterion. If their perfection was due to nearly parallel walls, straight instruments, adhesive gold, and the rubber dam, then all the other operations, when possible, must be performed in the same manner. This I accept as a general rule, though such a great variety of cavities occur that it will not do to generalize too freely.

What I most desire from gold is *strength* and *adaptability*. Strength I get from adhesive gold, and adaptability by cavities prepared to admit the use of nearly straight pluggers, and—backache! The strain upon the nervous system in packing adhesive gold is enormous, but I know of no "royal road" to permanent success of any kind. The gist of what I can say, then, on this subject of the consolidation of gold is, *small-pointed, nearly straight instruments, and the lead mallet in my own hands.* This for all cavities, large or small, when the rubber dam can be used; and it can nearly always be applied to all except the wisdom teeth,—and sometimes to the second molars before the wisdom teeth are erupted,—and in most cavities anterior to the second molars straight instruments can be used. I use the mallet myself that I may better control the force of the blow and better conduct the operation generally. Particularly do I find this true in contour fillings.

In packing gold by this method, of course I prepare my cavity with it in view.

I first decide from which side or from what direction I will fill, and then cut from that direction, as much as possible, with nearly straight chisels, so that when ready for filling, a straight plugger will reach every part of the cavity.

In approximal cavities of the incisors, for instance, I was taught, when a student, to pay little attention to the shape of the cervical wall, but to depend on the latteral walls and distal extremity for support. I reverse this now entirely. I leave no retaining-point at the distal extremity of the cavity, unless it is unavoidably so shaped by decay, and I can fill it with a straight instrument and mallet. I want the support for my filling in the base of the cavity, and along the lateral walls for a little distance from the base. With this in view, I excavate at the cervical wall until I get a firm foundation, leaving, if possible, no under-cut at the distal extremity. At the base I excavate in conformity with the naturally oval outline of the cavity, or cut the base at right angles with the lateral walls, as may seem best. In either case I drill a small retaining-point in the most acute angle of the base. This I do that I may mallet the first piece of gold firmly in the place where it belongs. Then, having both hands at liberty, I can carefully mallet every mat of gold that goes into the cavity. Proceeding in this way, I feel more sure of the foundation than by holding the first few mats in place and using only hand-pressure. These retaining-points are small, and are intended to give support to the gold only during the process of filling and not to the plug at large after the operation is completed.

A cavity prepared in this manner can, of course be filled with a single straight instrument, and the gold be consolidated entirely from one direction.

In packing the gold, I hold the plugger in the left hand—

which I seldom move from its support on the patient's face or chin,—and alternately introduce the gold, and use the mallet with the right. The gold I anneal, if necessary, and convey to its place in the cavity on the point of a small instrument used as a spear, or by delicately-pointed pliers. The points of our ordinary pliers are too large,—they rapidly absorb the heat from the flame and cause the gold to be unevenly annealed. Retaining this heat, they cause pain in applying the gold.

I pack the gold as full as I desire it to be, while going on with the operation, so that the last mat is put on at the distal extremity of the cavity. The foot-shaped instruments I use only for condensing the surface and margins, either during the process of filling or after the gold is all introduced. The burnisher I seldom use except at the margins—never after the file and stone. If the gold is not condensed by the plugger so as to file down perfectly smooth and free from pits or flaws, the burnisher cannot remedy its effects.

In approximal cavities in the incisors, when strength is not desired, I use gold No. 5, soft—it is called soft, and yet is sufficiently adhesive to pack well,—No. 4, adhesive, and Nos. 20 to 60, rolled. This latter I use a great deal, though not so much as formely, having learned from a pretty thorough trial where I can and where I cannot use it to advantage. The low numbers I fold in ribbon and cut to suit the case.

I fill in this manner all cavities that can be protected by the rubber dam, and that are sufficiently in the anterior part of the mouth to allow the use of straight instruments. Such parts of approximal cavities in the bicuspids and molars as cannot be easily reached by such instruments, I fill with pluggers bent at nearly a right angle, using the mouth-glass and hand-pressure.

The *exceptions* to this method are approximal cavities in the incisors, already so badly decayed as to render it impossible to prepare them without under-cuts that extend under thin walls; approximal cavities of the same kind in the bicuspids, and very often cavities in the grinding surfaces of molars and bicuspid, having small openings, and yet showing considerable decay interiorly. Such cavities I excavate thoroughly, and fill with oxychloride—more recently with Guillois' cement. After it has set, I cut portions of it away shaping the cavity as if decay had never gone beyond its margins, and fill with adhesive gold, packed, as before described, with straight instruments and the mallet.

This, I suppose, will be considered quite "orthodox" by some members of the profession,—especially those who are in the habit of using non-adhesive gold in cavities in the grinding surface. But if oxychloride can be left in a cavity where it has been put as a capping for an exposed pulp, why may it not be left in any part of a cavity where a straight plugger will not reach? That it will prevent decay we all very well know, and when protected from the action of the fluids of the mouth it seems to be the best material with which such cavities may be filled. It also has the advantage of being a non-conductor, so that nearly exposed pulps must be less endangered.

If oxychloride is too white to put under the enamel of incisors, Guillois' cement is darker and less objectionable than the yellow color of gold, and not so liable to discolor.

When the rubber dam cannot be used, and the operation must be performed quickly to avoid the fluids of the mouth, I know of nothing better than a sponge in approximal, and soft gold in grinding surface cavities, introduced and con densed by hand pressure, aided if possible, by the mallet. Operating as I do without an assistant, in such cases I use Salmon's automatic, the points being finely serrated, and patterned after those I use with the lead mallet, though somewhat larger. Such fillings are generally unsatisfactory, though of course better than none at all. I formerly operated almost entirely with the automatic, but found it having too much lateral motion, too liable to check the teeth from the sharpness of its blow, and not so obedient to the brain as one's own hand. In fact, it is too automatic.

Using oxychloride in badly-shaped, inaccessible cavities, it is unnecessary for me to say that I very seldom use cylinders.—*Dental Cosmos.*

---

